# Elevated brain glutamate levels in type 1 diabetes: correlations with glycaemic control and age of disease onset but not with hypoglycaemia awareness status

**DOI:** 10.1007/s00125-019-4862-9

**Published:** 2019-04-19

**Authors:** Evita C. Wiegers, Hanne M. Rooijackers, Jack J.A. van Asten, Cees J. Tack, Arend Heerschap, Bastiaan E. de Galan, Marinette van der Graaf

**Affiliations:** 1 Department of Radiology and Nuclear Medicine (766), Radboud university medical center, PO Box 9101, 6500 HB Nijmegen, the Netherlands; 2 Department of Internal Medicine, Radboud university medical center, Nijmegen, the Netherlands; 3 Department of Pediatrics, Radboud university medical center, Nijmegen, the Netherlands

**Keywords:** ^1^H MRS, Brain, Euglycaemia, Glutamate, Type 1 diabetes

## Abstract

**Aims/hypothesis:**

Chronic hyperglycaemia in type 1 diabetes affects the structure and functioning of the brain, but the impact of recurrent hypoglycaemia is unclear. Changes in the neurochemical profile have been linked to loss of neuronal function. We therefore aimed to investigate the impact of type 1 diabetes and burden of hypoglycaemia on brain metabolite levels, in which we assumed the burden to be high in individuals with impaired awareness of hypoglycaemia (IAH) and low in those with normal awareness of hypoglycaemia (NAH).

**Methods:**

We investigated 13 non-diabetic control participants, 18 individuals with type 1 diabetes and NAH and 13 individuals with type 1 diabetes and IAH. Brain metabolite levels were determined by analysing previously obtained ^1^H magnetic resonance spectroscopy data, measured under hyperinsulinaemic–euglycaemic conditions.

**Results:**

Brain glutamate levels were higher in participants with diabetes, both with NAH (+15%, *p* = 0.013) and with IAH (+19%, *p* = 0.003), compared with control participants. Cerebral glutamate levels correlated with HbA_1c_ levels (*r* = 0.40; *p* = 0.03) and correlated inversely (*r* = −0.36; *p* = 0.04) with the age at diagnosis of diabetes. Other metabolite levels did not differ between groups, apart from an increase in aspartate in IAH.

**Conclusions/interpretation:**

In conclusion, brain glutamate levels are elevated in people with type 1 diabetes and correlate with glycaemic control and age of disease diagnosis, but not with burden of hypoglycaemia as reflected by IAH. This suggests a potential role for glutamate as an early marker of hyperglycaemia-induced cerebral complications of type 1 diabetes.

ClinicalTrials.gov NCT03286816; NCT02146404; NCT02308293

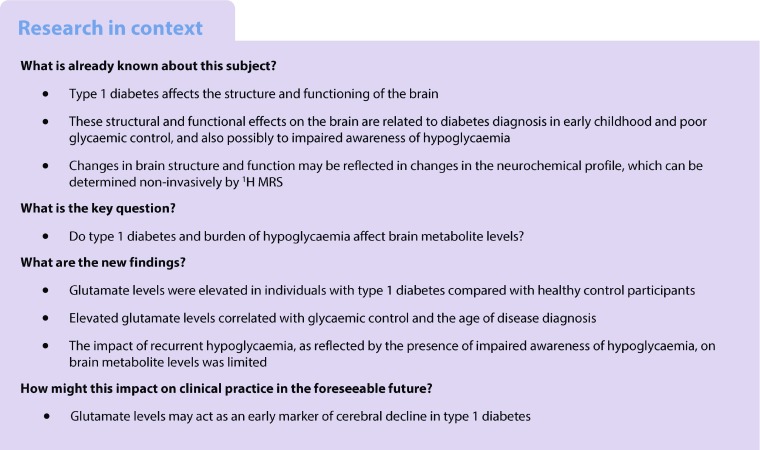



## Introduction

Type 1 diabetes has a negative effect on the structure and functioning of the brain [1]. Several studies report on a lower cognitive performance in people with type 1 diabetes compared with non-diabetic individuals, particularly regarding memory function and learning [2–4]. Structural cerebral changes, such as a reduced grey matter volume, have also been described in type 1 diabetes [5–7]. Moreover, it is known that type 1 diabetes affects cerebral metabolism and metabolic pathways [8], especially in response to acute hypoglycaemia [9]. Known risk factors for these structural and functional effects on the brain include diabetes onset in early childhood [10, 11] and poor glycaemic control [12]. Recently, the presence of impaired awareness of hypoglycaemia (IAH), which results from exposure to recurrent hypoglycaemia, has also been associated with reduced cognitive function [13] and structural cerebral decline [5]. The neurochemical mechanism(s) that may precede, underlie or accompany these effects on the brain are not well studied, hence elucidation of such mechanism may provide further insight into detrimental effects of type 1 diabetes on the brain.

^1^H MR spectroscopy (MRS) is a suitable tool for the non-invasive examination of a range of neuro-metabolites in humans [14]. Previous studies using ^1^H MRS have suggested that a change in brain glutamate may have a role in neuronal function loss in type 1 diabetes. Under hyperglycaemic conditions, the levels of glutamate and *N*-acetylaspartate (NAA) were lower in individuals with long-term type 1 diabetes compared with non-diabetic control participants [15]. Since NAA and glutamate are metabolites that mainly reside in neurons, these lower levels were attributed to a loss of neuronal function. However, such lower levels were not found when individuals were studied under euglycaemic conditions [16]. Moreover, in a large cohort, an increase in prefrontal glutamate levels in type 1 diabetes was described, with a linear relation between glutamate levels and lifetime glycaemic control [4]. The impact of recurrent hypoglycaemia on brain metabolite levels is currently not known.

Using IAH as a proxy for significant burden of hypoglycaemia, we aimed to assess the impact of recurrent hypoglycaemia on brain metabolites as measured by ^1^H MRS by comparing these in individuals with type 1 diabetes mellitus and IAH to those with normal awareness of hypoglycaemia (NAH) and non-diabetic control participants. Furthermore, we aimed to evaluate the effect of potential mediators for structural and functional cerebral decline, such as level of glycaemic control and age at diagnosis, on metabolites in the brain.

## Methods

The ^1^H MRS data were acquired as part of three previously conducted studies [17–19], focusing on cerebral lactate levels during euglycaemia and hypoglycaemia, using the hyperinsulinaemic clamp technique. Here we analysed the total neurochemical profile of the participants as seen by ^1^H MRS, recorded under hyperinsulinaemic–euglycaemic conditions. These data had not been previously analysed. Details regarding the hyperinsulinaemic glucose clamp have been reported previously [17–19]. Participants were not subjected to any intervention other than the hyperinsulinaemic–euglycaemic clamp during data acquisition. All data were acquired between September 2014 and August 2017 and all participants gave written informed consent. The studies were approved by and studied in accordance with the ethical standards of the institutional review board of the Radboud university medical center (Commissie Mensgebonden Onderzoek Arnhem-Nijmegen) and with the Declaration of Helsinki of 1975/1983 and its later amendments and revisions.

The cohort consisted of 13 individuals without diabetes, 18 with type 1 diabetes and NAH, and 13 with type 1 diabetes and IAH. Participants with type 1 diabetes were initially classified as NAH or IAH using the Dutch modified version of the Cox questionnaire [20, 21], and their state of awareness was retrospectively confirmed during clamped hypoglycaemia on the basis of adrenaline and hypoglycaemic symptom responses. HbA_1c_ levels of the individuals with type 1 diabetes were determined at the time of inclusion. Participants with type 1 diabetes were further subdivided into those with optimal glycaemic control (i.e. HbA_1c_ <53 mmol/mol [7.0%]) and suboptimal glycaemic control (i.e., HbA_1c_ ≥53 mmol/mol [7.0%]). Main exclusion criteria were: MRI contraindications, the use of drugs (other than insulin) that may alter glucose metabolism, HbA_1c_ levels >75 mmol/mol (9.0%) and presence of microvascular complications, except for background retinopathy.

### MRS protocol and data processing

MRS measurements were performed on a 3T MR system (Tim MAGNETOM Trio (*n* = 32) or MAGNETOM Prisma-fit (*n* = 12), Siemens, Erlangen, Germany), using a body coil for excitation and a 12-channel receive-only head coil. A T_1_-weighted anatomical image (MPRAGE; 256 × 256 mm^2^ field of view; 1 mm^3^ isotropic voxels) was used for MRS voxel positioning and for determination of the amount of grey matter, white matter and cerebrospinal fluid (CSF) in the MRS voxel.

^1^H MRS data were acquired from a single voxel (22.5–25.0 cm^3^) in the periventricular region of the brain. A semi-LASER spectroscopy sequence [22] was used with an 8-step phase cycle and an echo time (TE) of 30 ms (Trio) or 33 ms (Prisma-fit), a repetition time (TR) of 3000 ms and 32 averages, combined with WET water suppression (water suppression enhanced through T_1_ effects) [23]. A water-unsuppressed spectrum was acquired for each participant from the same voxel with a TR of 5000 ms, a TE of 30 or 33 ms and 4 averages, used for eddy-current correction and for absolute metabolite quantification.

Spectra were analysed with LCModel software [24]. The LCModel basis set, one for each TE, was created in Bruker TopSpin (Bruker, Billerica, MA, USA) and contained 19 metabolites: Ala, Asp, choline, creatine, γ-aminobutyric acid (GABA), glucose, Glu, Gln, glutathione, glycerophosphocholine, Gly, lactate, *myo*-inositol, NAA, *N*-acetylaspartylglutamate (NAAG), phosphocholine, phosphocreatine, *scyllo*-inositol and taurine. The basis set was extended with a previously measured macromolecular baseline [25]. The spectral region between 0.5 and 4.2 ppm was used in the LCModel analysis. Metabolites quantified with a Cramér–Rao lower bound (CRLB) >50% were classified as undetected and only metabolites quantified with a CRLB <50% in at least half of the spectra were used in the analysis. The CRLB cut-off of 50% was chosen to avoid direct rejection of metabolites with a low concentration [26]. If the covariance between metabolites was consistently high (i.e., *r* below −0.3), the sum of the related metabolites was reported; this was the case for total NAA (NAA + NAAG), total choline (phosphocholine + glycerophosphocholine) and total creatine (creatine + phosphocreatine), but not for Glu + Gln (mean *r*: 0.02 ± 0.14). The signal-to-noise ratio (SNR) and spectral linewidths were analysed in the context of quality assessment. SNR was defined as the ratio of the maximum height of the largest signal (i.e., NAA) to twice the root-mean-square of the noise and the spectral linewidth as the full width at half-maximum of the respective water-unsuppressed spectrum. The SNR and linewidths were reported by LCModel.

To determine the relative proportions of grey matter, white matter and CSF in the MRS voxel, we segmented the T_1_-weighted images using Matlab 2017b (MathWorks, Natick, MA, USA) and the VBM8 toolbox in SPM8 (Functional Imaging Laboratory, University College London, London, UK). These fractions were used to estimate the water content in the MRS voxel, assuming a water concentration of 43.3 mol/l in grey matter, 35.9 mol/l in white matter and 55.6 mol/l in CSF [27], and to correct for partial volume effects [28]. Concentrations were also corrected for T_1_ and T_2_ relaxation.

#### Statistical analysis

Differences in metabolite levels between individuals with IAH, those with NAH and non-diabetic control participants were determined using ANOVA with Bonferroni post hoc tests to accommodate the three groups design. Analyses were repeated using ANCOVA to adjust comparisons for voxel content (i.e., the amount of grey matter in the voxel). ANOVA and ANCOVA results were similar; therefore only those calculated using ANOVA are reported.

Linear regression analysis was performed between glutamate levels and HbA_1c_ levels and between glutamate levels and the age at diagnosis of diabetes. In a secondary analysis we subdivided the participants into those with optimal or suboptimal glycaemic control. ANOVAs were conducted to detect differences between non-diabetic individuals, individuals with NAH and optimal glycaemic control (mean HbA_1c_: 48.2 ± 2.9 mmol/mol [6.5 ± 0.3%], *n* = 6), those with NAH and suboptimal glycaemic control (59.7 ± 4.8 mmol/mol [7.6 ± 0.4%), *n* = 12], those with IAH and optimal glycaemic control (47.6 ± 7.7 mmol/mol [6.5 ± 0.7%], *n* = 5) and those with IAH and suboptimal glycaemic control (58.6 ± 4.9 mmol/mol [7.5 ± 0.4%], *n* = 8).

Data are presented as mean ± SD. All statistical analyses were performed in IBM SPSS Statistics 22 (IBM, Chicago, IL, USA). A *p* value less than 0.05 was considered statistically significant. No correction for multiple comparisons was performed.

## Results

The participants were matched for age, sex and BMI, and for diabetes duration and HbA_1c_ levels in the patient groups (Table 1). There were also no significant differences in these variables (except for HbA_1c_ levels) between the subgroups of participants subdivided according to glycaemic control (data not shown). Plasma glucose levels were well in the euglycaemic range during the acquisition of the MRS data, with no differences between groups (overall mean: 5.3 ± 0.6 mmol/l and a mean CV of 10.6%).

**Table 1:** Participant characteristics

Variable	Non-diabetic control (*n* = 13)	T1DM NAH (*n* = 18)	T1DM IAH (*n* = 13)
Age (years)	25.5 ± 5.5	25.3 ± 7.2	26.1 ± 6.8
Sex (male/female)	6 / 7	10 / 8	7 / 6
BMI (kg/m^2^)	23.5 ± 1.6	24.0 ± 2.6	23.7 ± 1.5
Duration of T1DM (years)		11.4 ± 6.8	14.1 ± 9.3
HbA_1c_ (mmol/mol)		55.8 ± 6.9	54.4 ± 8.0
HbA_1c_ (%)		7.3 ± 0.6	7.1 ± 0.7

Data are presented as number or mean ± SD

T1DM IAH, type 1 diabetes with IAH; T1DM NAH, type 1 diabetes with NAH

**Fig. 1:**
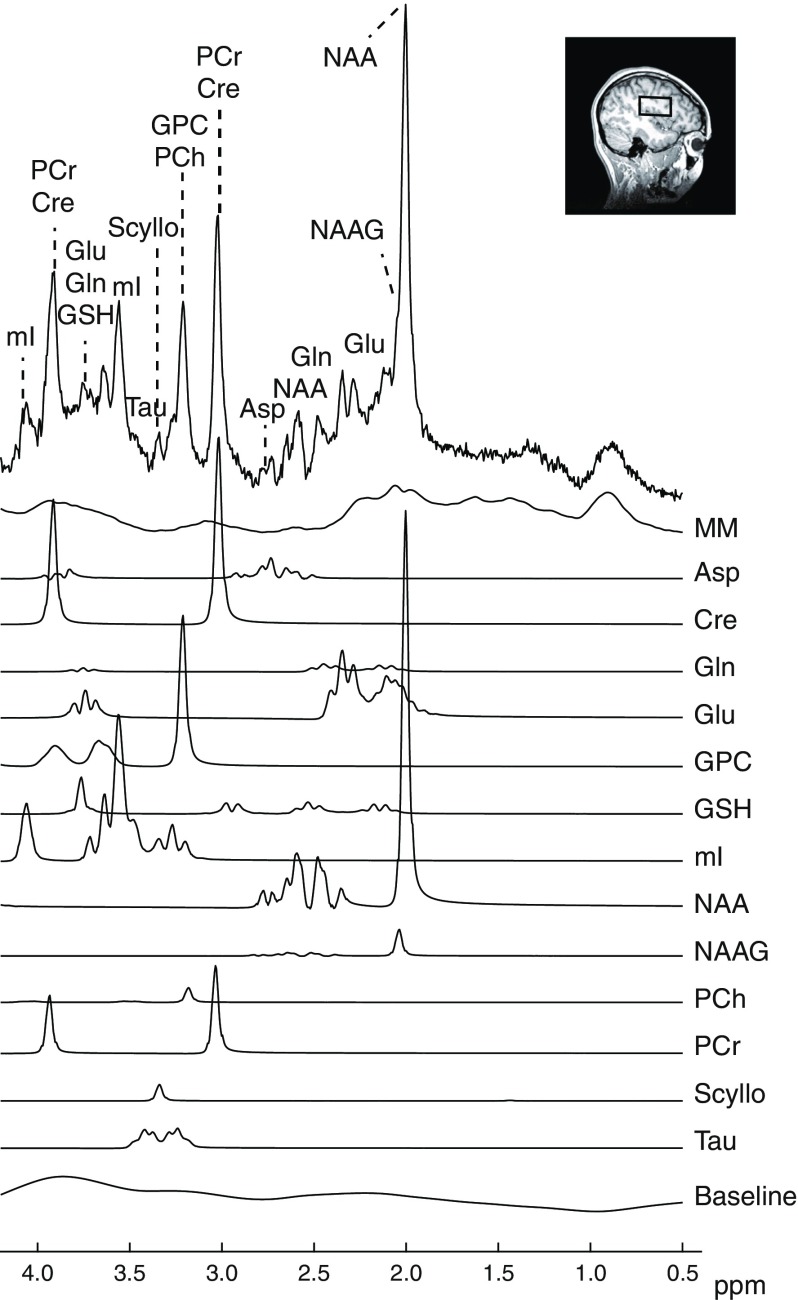
Example of LCModel analysis of one ^1^H MR spectrum (top row). The peaks identified include macromolecules (MM), aspartate (Asp), creatine (Cre), glutamine (Gln), glutamate (Glu), glycerophosphocholine (GPC), glutathione (GSH), *myo*-inositol (mI), *N*-acetylaspartate (NAA), *N*-acetylaspartylglutamate (NAAG), phosphocholine (PCh), phosphocreatine (PCr), *scyllo*-inositol (Scyllo) and taurine (Tau). The insert shows the localisation of the MRS voxel

The ^1^H MR spectra were of good quality (see Fig. 1), with comparable linewidths (overall mean: 0.05 ± 0.01 ppm) and SNR (overall mean: 39.7 ± 7.9) between groups. The MRS voxel contained on average ~65–70% (parietal) white matter, with no differences in voxel composition between groups (Table 2).

**Table 2:** SNR, linewidths and voxel composition

Variable	Non-diabetic control	T1DM NAH	T1DM IAH	*p* value
SNR	41.2 ± 6.9	40.5 ± 7.3	36.9 ± 9.5	0.34
Linewidth (ppm) [Hz]	0.05 ± 0.01 [6.5 ± 1.4]	0.05 ± 0.01 [6.3 ± 0.9]	0.05 ± 0.01 [6.0 ± 0.9]	0.54
Grey matter (%)	26.5 ± 4.2	29.5 ± 6.0	31.1 ± 6.3	0.12
White matter (%)	71.3 ± 4.7	67.6 ± 6.9	66.0 ± 7.1	0.11
CSF (%)	2.2 ± 0.9	2.9 ± 1.3	3.1 ± 1.4	0.18

Data are presented as number or mean ± SD

T1DM IAH, type 1 diabetes with IAH; T1DM NAH, type 1 diabetes with NAH

Ten metabolites were consistently quantified using LCModel. Mean CRLBs were <10% for Glu, glutathione, *myo*-inositol, total creatine, total choline and tNAA and <30% for Asp, Gln, *scyllo*-inositol and taurine (Table 3).

**Table 3:** Quantification results

	Metabolite										
	Asp	Gln	Glu	GSH	mI	Scyllo	Tau	tCho	tCre	tNAA	MM
Healthy control participants											
Mean (μmol/g, ww)	2.07	1.32	6.10	0.94	5.51	0.57	1.74	1.79	6.94	11.12	48.30
SD (μmol/g, ww)	0.34	0.44	0.74	0.12	0.79	0.17	0.50	0.16	0.41	0.55	9.36
Mean CRLB (%)	20.9	26.8	5.5	8.4	4.5	17.1	17.2	3.8	2.0	1.7	11.6
SD CRLB (%)	4.9	6.7	0.9	1.0	1.1	5.9	6.0	2.5	0.0	0.4	3.5
*n*	13	12	13	13	13	13	12	13	13	13	13
Type 1 diabetes and NAH											
Mean (μmol/g, ww)	2.26	1.45	7.04	1.02	5.45	0.41	1.98	1.90	6.92	10.74	45.10
SD (μmol/g, ww)	0.51	0.57	0.86	0.13	1.09	0.13	0.54	0.39	0.51	0.58	7.20
Mean CRLB (%)	20.8	27.2	5.1	8.6	4.8	23.7	17.7	3.3	2.0	1.7	13.1
SD CRLB (%)	8.8	8.9	0.8	1.6	2.0	6.5	6.7	1.6	0.0	0.5	3.7
*n*	17	18	18	18	18	17	17	18	18	18	18
Type 1 diabetes and IAH											
Mean (μmol/g, ww)	2.70	1.64	7.29	1.22	5.86	0.45	1.63	1.97	7.07	10.98	48.29
SD (μmol/g, ww)	0.61	0.68	0.93	0.57	0.93	0.18	0.39	0.31	0.44	0.46	14.31
Mean CRLB (%)	18.2	25.2	5.5	8.5	4.4	24.1	24.1	3.8	2.1	1.9	13.5
SD CRLB (%)	5.1	6.7	1.2	2.4	1.3	8.2	8.2	3.0	0.4	0.6	8.0
*n*	13	13	13	13	13	12	13	13	13	13	13

GSH, glutathione; mI, *myo*-Inositol; MM, macromolecules; Scyllo, *scyllo*-inositol; Tau, taurine; tCho, total choline; tCre, total creatine; tNAA, total NAA; ww, wet weight

**Fig. 2:**
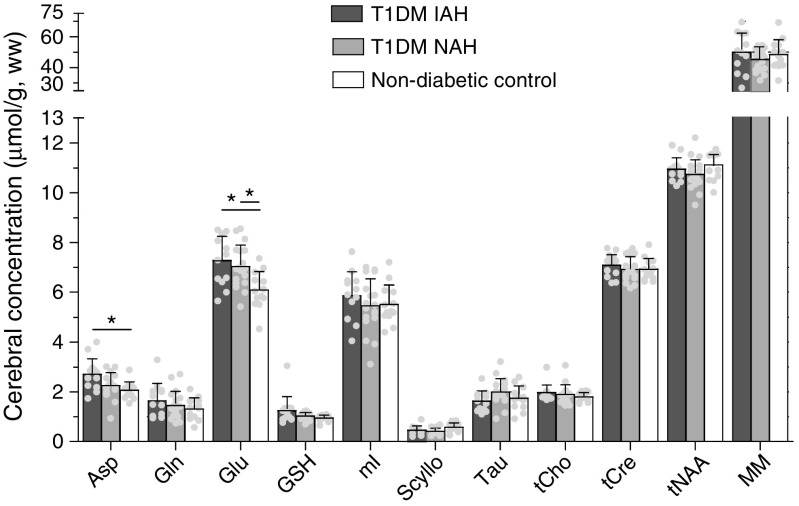
Mean metabolite levels in participants with type 1 diabetes and IAH (T1DM IAH), participants with type 1 diabetes and NAH (T1DM NAH) and non-diabetic control participants. Between group differences were assessed with ANOVA with Bonferroni post hoc tests to accommodate the three groups design; *p < 0.05. Data are mean + SD, with individual data points shown as dots. GSH, glutathione; mI, *myo*-inositol; MM, macromolecules; Scyllo, *scyllo*-inositol; Tau, taurine; tCho, total choline; tCre, total creatine; tNAA, total NAA; ww, wet weight

Brain glutamate levels were significantly higher in individuals with type 1 diabetes, both in those with NAH (+15%, *p* = 0.013) and in those with IAH (+19%, *p* = 0.003), when compared with non-diabetic control participants. Glutamate levels did not differ between the two patient subgroups (*p* = 1.0). Furthermore, we observed higher aspartate levels in type 1 diabetes and IAH (+30%, *p* = 0.009) compared with non-diabetic control participants. All other metabolite levels, as well as the level of macromolecules, were similar across groups (Fig. 2).

**Fig. 3:**
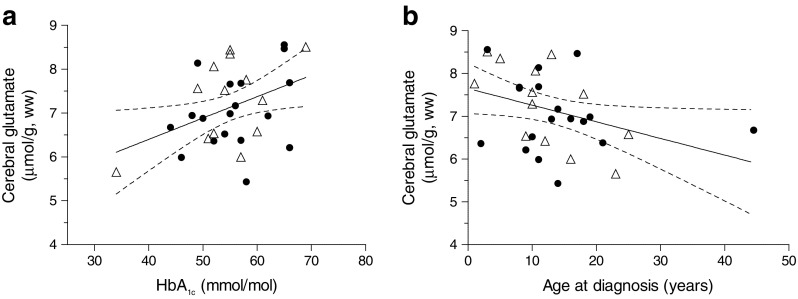
Correlation between cerebral glutamate levels and HbA_1c_ levels (**a**) and between cerebral glutamate levels and the age at diagnosis of type 1 diabetes (**b**). Data from participants with type 1 diabetes and IAH (T1DM IAH; white triangles) and participants with type 1 diabetes and NAH (T1DM NAH; black circles), together with the linear fits of the data and the 95% CI, obtained from linear regression analysis. (**a**) *p =* 0.03; *r* = 0.40; (**b**) *p* = 0.04; *r* = −0.36; ww, wet weight

**Fig. 4:**
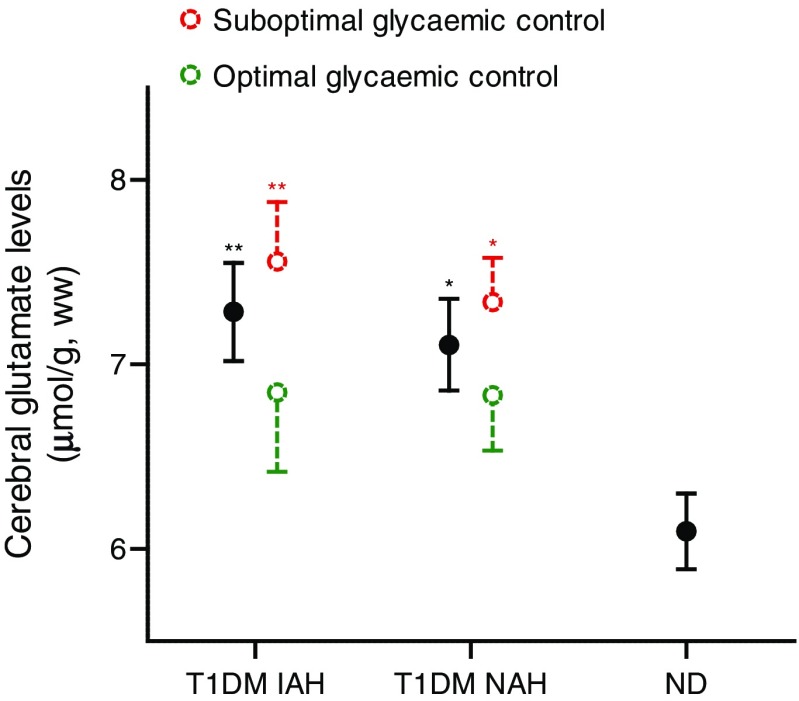
Cerebral glutamate levels. Group means are depicted in black circles. Participants with type 1 diabetes are further subdivided into those with optimal glycaemic control (HbA_1c_ <53 mmol/mol [7.0%]) and those with suboptimal glycaemic control (HbA_1c_ ≥53 mmol/mol). ANOVAs were conducted to detect differences among non-diabetic individuals, and diabetic individuals with NAH and optimal glycaemic control, NAH and suboptimal glycaemic control, IAH and optimal glycaemic control, and IAH and suboptimal glycaemic control; *p < 0.05, **p < 0.01 vs non-diabetic control participants. ND, non-diabetic control; T1DM, type 1 diabetes; ww, wet weight

Cerebral glutamate levels were linearly correlated to HbA_1c_ levels in individuals with type 1 diabetes (*p =* 0.03; *r* = 0.40; Fig. 3a) and inversely correlated to the age at diagnosis of diabetes (*p* = 0.04; *r* = −0.36; Fig. 3b). The variance inflation factor (VIF) between both predictors was 1.19, meaning that there was no noteworthy collinearity within our data. A sensitivity analysis excluding outliers did not materially change these associations. There was no correlation between cerebral glutamate levels and the duration of type 1 diabetes. Subdividing the participants into those with optimal or suboptimal glycaemic control gave further insight into the elevated cerebral glutamate levels. Cerebral glutamate levels were particularly elevated in participants with suboptimal glycaemic control, and particularly in those with IAH (Fig. 4). There were no differences in glutamate levels between both subgroups of individuals with optimal glycaemic control and non-diabetic individuals (Fig. 4). There was no difference between male and female participants.

## Discussion

Here we evaluated the neurochemical profile of participants with type 1 diabetes, with and without IAH, under euglycaemic conditions and compared this with non-diabetic control participants. Our main finding was that brain glutamate levels are elevated in both groups of participants with diabetes compared with healthy non-diabetic control participants. Known risk factors for functional and structural cerebral decline in type 1 diabetes, i.e. glycaemic control and an early age of disease onset, are correlated to these elevated glutamate levels. The presence or absence of IAH did not affect glutamate levels.

Glutamate is one of the most important excitatory neurotransmitters and studies have shown that increased glutamate levels are roughly linearly related with increased excitatory activity [29]. Therefore, changes in glutamate levels have been linked to metabolic activity [29, 30]. The greater cerebral glutamate levels in people with type 1 diabetes as compared with those without diabetes could be interpreted as an upregulation of cerebral metabolism in individuals with type 1 diabetes. However, this contrasts with studies that reported no significant difference in the tricarboxylic acid (TCA) cycle rate during euglycaemia between individuals with type 1 diabetes and non-diabetic control participants [31]. Alternatively, glutamate itself may act as a metabolic substrate for the TCA cycle, through conversion to α-ketoglutarate. Previous studies have shown that cerebral glutamate levels drop in response to acute hypoglycaemia in individuals with type 1 diabetes with intact hormonal responses to hypoglycaemia, which was attributed to glutamate acting as an alternative fuel [18, 19, 32]. Glutamate oxidation can complement glucose use [33]. The higher glutamate levels could be the result of a cerebral protection mechanism in response to multiple hypoglycaemic insults, and thus cerebral glucose deprivation, during a lifetime with diabetes. Finally, elevated extracellular glutamate levels are known to be excitotoxic, which triggers various processes that result in neuronal cell death. Glutamate excitotoxicty is thought to be involved in neurodegenerative diseases such as Parkinson’s disease and Huntington’s disease [34], which also show higher cerebral glutamate levels [35]. However, since the extracellular level of glutamate in the brain is only a few μmol/l, it is not expected to be detected by ^1^H MRS.

Our results are in line with a large cohort study in which elevated prefrontal glutamate levels were found in individuals with long-standing type 1 diabetes [4]. These higher glutamate concentrations were associated with lower cognitive function as well as with mild depression, and correlated with lifetime glycaemic control. Here we show that another risk factor for cerebral decline, namely the age of onset of diabetes, also correlated with cerebral glutamate levels. These findings indicate that elevated brain glutamate may be an early marker of the potentially devastating effect of chronic hyperglycaemia, in particular on the developing brain. The presence of IAH alone has only limited additional consequences regarding cerebral glutamate levels. Interestingly, glutamate levels were the highest, although this difference was not significant, in individuals with both IAH and suboptimal HbA_1C_ levels, which may suggest that individuals with the most fluctuating glucose levels are more prone to these alterations.

Our data contrasts with a ^1^H MRS study that showed a decline in cerebral glutamate levels in people with type 1 diabetes [15]. In that study, however, data were acquired during a hyperglycaemic clamp, which may have altered cerebral metabolism [36, 37]. Furthermore, these lower glutamate levels were only found in a grey-matter-rich voxel and not in a white-matter-rich region as studied here, suggestive of region-specific metabolic alterations, as also described in a rat model of type 1 diabetes [38].

We also found increased aspartate levels in participants with IAH compared with non-diabetic control participants. Aspartate, like glutamate, is an excitatory neurotransmitter and both are synthesised from glucose in the brain. Aspartate and glutamate are also linked through the malate-aspartate shuttle (MAS). However, an increased flux through the MAS implies opposite changes in glutamate and aspartate levels [30], which is not in line with our results.

Our finding of increased glutamate levels in the brain of individuals with type 1 diabetes is also important in the context of studies on brain metabolism with ^13^C MRS and metabolic modelling. Brain glutamate levels are typically used as an input in such models and often it is assumed that glutamate levels are constant (e.g. no change upon hypoglycaemia) and not different between different groups of individuals (e.g. no difference between individuals with and without diabetes) [31, 39]. These assumptions are critical and deviations may have a significant impact on outcomes. The results of the current study presents an argument for using measured glutamate levels as input in such models.

The present study has some limitations. MRS data were acquired from a large single voxel, which precludes any analysis of the regional dependency of the described alterations. Furthermore, we performed an explorative analysis on multiple metabolites in a rather limited sample size. As a consequence of clamp conditions, insulin levels were elevated albeit within the physiological range. We are, however, unaware of modulating effects of insulin on brain glutamate levels. Furthermore, we used the presence of IAH as proxy for recurrent hypoglycaemia, since habituation to frequent hypoglycaemia underlies the pathogenesis of IAH. We acknowledge that IAH is not an on/off phenomenon, so an impact of hypoglycaemia, which also occurs in individuals with NAH, cannot be fully excluded. Also, we did not actually measure burden of hypoglycaemia, but based on the pathogenesis of IAH we assumed this burden to be greater in people with IAH than in those with NAH. Finally, inherent to the inclusion criteria, the participants with type 1 diabetes all had HbA_1c_ levels below 9.0% and were relatively young. Future research should focus on the effect of more poorly controlled and long-standing diabetes.

In conclusion, we show that there are alterations in neuro-metabolites in individuals with type 1 diabetes. Cerebral glutamate levels are higher in type 1 diabetes and correlate with known risk factors for cerebral decline, suggesting a potential role for glutamate as an early indicator of cerebral complications of type 1 diabetes. The presence of IAH, which usually results from recurrent hypoglycaemia, has only limited additional impact on the neurochemical profile of individuals with type 1 diabetes.

## Data Availability

Data may be obtained from the corresponding author on request.

## Abbreviations

CRLB: Cramér–Rao lower bound
CSF: Cerebrospinal fluid
IAH: Impaired awareness of hypoglycaemia
MAS: Malate-aspartate shuttle
MRS: Magnetic resonance spectroscopy
NAA: *N*-acetylaspartate
NAAG: *N*-acetylaspartylglutamate
NAH: Normal awareness of hypoglycaemia
SNR: Signal-to-noise ratio
TCA: Tricarboxylic acid
TE: Echo time
TR: Repetition time
